# Photoelectrocatalytic Degradation of Rhodamine B in the Presence of TiO_2_-BiVO_4_

**DOI:** 10.3390/ma18184253

**Published:** 2025-09-11

**Authors:** Anli Sun, Chao Kong, Jie Wang, Beihai Zhou, Huilun Chen, Rongfang Yuan, Zhiming Bai

**Affiliations:** 1 Beijing Key Laboratory of Resource-Oriented Treatment of Industrial Pollutants, School of Energy and Environmental Engineering, University of Science and Technology Beijing, Beijing 100083, China; m202320235@xs.ustb.edu.cn (A.S.); kongchao@ceri.com.cn (C.K.); m202310186@xs.ustb.edu.cn (J.W.); zhoubeihai@sina.com (B.Z.); chenhuilun@ustb.edu.cn (H.C.); 2 School of Resources and Safety Engineering, University of Science and Technology Beijing, Beijing 100083, China; baizhiming2008@126.com

**Keywords:** rhodamine B, photoelectrochemical performance, TiO_2_-BiVO_4_, composite/layered heterostructure, degradation pathways

## Abstract

The discharge of printing and dyeing wastewater has become a key concern in global water pollution control due to its high pollutant concentration, dark color, refractory biodegradability and toxic characteristics. Photoelectrocatalytic (PEC) technology has gained widespread attention as it can effectively treat refractory organic pollutants. In this study, titanium dioxide (TiO_2_)–bismuth vanadate (BiVO_4_) composite materials were synthesized through the sol–gel/solvothermal hybrid method, and layered heterojunction structures were fabricated via sol–gel precursor preparation followed by spin-coating deposition. The PEC degradation efficiency of rhodamine B (RhB) was systematically evaluated under varying operational conditions in the presence of TiO_2_-BiVO_4_. The four-layer BiVO_4_/four-layer TiO_2_ material showed the optimal catalytic activity among the tested structures, achieving an 80.3% removal of RhB under an applied bias of 4 V and illumination intensity of 14,000 lx. Through the equilibrium adjustment of the Fermi levels, the type Ⅱ heterostructure was formed. Moreover, superoxide radical (O_2_^−^) was identified as the predominant reactive oxygen species driving the degradation mechanism. Mechanistic analysis revealed that RhB degradation was accomplished through deethylation, benzene ring cleavage, and subsequent ring-opening mineralization. This study prepared an efficient PEC material, which provides a theoretical basis for the PEC treatment of printing and dyeing wastewater.

## 1. Introduction

The printing and dyeing industry, a cornerstone of global textile manufacturing, has expanded significantly alongside economic growth and rising living standards. This expansion has correspondingly amplified wastewater discharge containing persistent synthetic dyes. These dyes and auxiliaries demonstrate remarkable persistence, with high salinity impairing biological treatment efficiency and extreme pH values damaging aquatic life and treatment infrastructure [1,2]. Additionally, the diversity of printing and dyeing processes and fiber types creates substantial wastewater quality variability [3]. Rhodamine B (RhB) is a fluorescent xanthene dye with high water/ethanol solubility and exceptional chemical stability [4]. Characterized by its oxygen-bridged aromatic structure, RhB resists degradation while exhibiting toxic effects through bioaccumulation in aquatic ecosystems. Its light absorption characteristics exacerbate water discoloration, which impairs photosynthesis by reducing sunlight penetration. Moreover, its ability to chelate metal ions can induce chronic toxicity in aquatic organisms [5]. Traditional methods like biodegradation and adsorption can partially treat these effluents, but remain challenged by low efficiency, high costs, and secondary pollution risks.

Photoelectrocatalysis (PEC) is an emerging technology that integrates photocatalysis (PC) and electrochemistry (EC). By utilizing semiconductors as electrodes and incorporating light fields into the EC process, PEC achieves synergistic photoelectric catalysis, thereby enhancing photocarrier productivity and facilitating redox reactions [6]. In recent years, PEC technology, as a sustainable and eco-friendly technology, has been widely applied in H_2_ production, CO_2_ reduction, and NH_3_ synthesis [7,8,9]. Additionally, PEC is effective in treating wastewaters with low biodegradability, high complexity, and high concentration of pollutants [10,11].

The selection of photoelectrode materials constitutes a critical determinant in photoelectrocatalytic (PEC) system design. Titanium dioxide (TiO_2_) and bismuth vanadate (BiVO_4_) have emerged as two prominent semiconductor candidates with distinct operational characteristics. TiO_2_ has been extensively employed in pollutant degradation due to its exceptional physicochemical stability and favorable charge transfer properties [12]. Nevertheless, its practical implementation faces two principal constraints. Firstly, rapid recombination of photogenerated electron–hole (e^−^/h^+^) pairs substantially diminishes catalytic efficiency [13]. Secondly, its wide bandgap (~3.2 eV) restricts optical absorption to ultraviolet wavelengths (<400 nm), thereby failing to harness the visible light spectrum (400–760 nm), which represents approximately 45% of incident solar energy [14]. In comparison, monoclinic scheelite-phase BiVO_4_ demonstrates enhanced solar energy conversion capabilities through its narrower 2.4 eV bandgap [15,16]. However, this bismuth-based semiconductor encounters analogous charge carrier limitations, particularly inefficient separation of conduction band e^−^ and valence band h^+^, compounded by persistent carrier recombination phenomena that collectively degrade its practical catalytic performance [17].

To address these challenges, modification strategies have been developed to synergistically enhance charge separation dynamics and light utilization. Among them, the construction of heterojunction interfaces has emerged as a particularly effective solution. By establishing an interfacial electric field to directionally drive photogenerated e^−^/h^+^ separation and broadening the light absorption range, photocatalytic performance is thereby significantly improved [18]. For instance, Adhikari et al. deposited Fe_2_O_3_ onto Bi_2_WO_6_ to construct heterojunction photoelectrodes for PEC sensing and degradation of tetracycline (TC) [19]. In general, the design and preparation of composite semiconductor materials can be achieved by selecting components, optimizing interface properties, and adjusting size, among other factors. Currently, photoelectrodes formed from these composite materials are widely employed in the PEC field.

The performance of PEC is significantly influenced by various parameters. In addition to the type of photoelectrode and PEC reactor design, operating conditions, such as the external potential, current density applied to the photoelectrode, and light source intensity, are decisive factors influencing PEC performance [20,21]. In addition, water quality parameters, including solution pH, coexisting ions, and electrolyte concentration, also play a crucial role in PEC performance [22,23,24]. By studying the influence of various factors, the PEC system can be optimized to address the ongoing environmental pollution.

In this study, two distinct TiO_2_-BiVO_4_ heterojunction materials were developed. The preparation process of TiO_2_-BiVO_4_ materials was optimized based on RhB removal efficiency, with subsequent parameter adjustments under varying operational conditions (e.g., applied bias voltage, light intensity, and electrolyte composition) to maximize catalytic activity. The chemical composition, optical properties, pore structure, and morphology of the synthesized materials were systematically characterized. The recyclability and long-term stability of the materials were rigorously assessed to determine their practical applicability. Additionally, the catalytic mechanism of TiO_2_-BiVO_4_ and the degradation pathway of RhB in the PEC system were elucidated through energy band alignment and identification of reactive oxygen species. This work provides basic support for the development of efficient PEC materials for environmental applications.

## 2. Materials and Methods

### 2.1. Materials

Glacial acetic acid (CH_3_COOH), sodium sulfate (Na_2_SO_4_) and sodium nitrate (NaNO_3_) (all AR grade) from MaxScientific Inc. (Beijing, China). Hydrochloric acid (HCl) (AR) from Tianjin Guangfu Technology Development Co., Ltd. (Tianjin, China). Ethanol absolute (C_2_H_5_OH) and acetone (C_3_H_6_O) (both AR) from Beijing Chemical Works (Beijing, China). Bismuth nitrate pentahydrate (Bi(NO_3_)_3_·5H_2_O) and ammonium vanadate (NH_4_VO_3_) (both AR) from Beijing HongHu LianHe HuaGong ChanPin Co., Ltd. (Beijing, China). Isopropanol (IPA, C_3_H_8_O), *p*-benzoquinone (BQ, C_6_H_4_O_2_) (both AR) and EDTA-2Na (C_10_H_14_N_2_Na_2_O_8_, 98%) from Shanghai Macklin Biochemical Technology Co., Ltd. (Shanghai, China). RhB (C_28_H_31_ClN_2_O_3_), tetrabutyl orthotitanate (TBT, C_16_H_36_O_4_Ti), titanium tetraisopropanolate (C_12_H_28_O_4_Ti), nitric acid (HNO_3_) and sodium hydroxide (NaOH) (all AR) from Sinopharm Chemical Reagent Co., Ltd. (Shanghai, China).

### 2.2. Preparation of TiO_2_-BiVO_4_ Composite and Layered Materials

The TiO_2_-BiVO_4_ composite (TiO_2_-BiVO_4_-M) and layered (TiO_2_-BiVO_4_-L) materials were synthesized through a combination of sol–gel-hydrothermal and spin-coating methods. To prepare BiVO_4_ precursors, 4.85 g Bi(NO_3_)_3_·5H_2_O was dissolved in 5 mL HNO_3_ (16 mol·L^−1^), diluted to 20 mL with deionized water, and stirred for 1 h. Concurrently, 1.17 g NH_4_VO_3_ was dissolved in 20 mL NaOH (4 mol·L^−1^) under 30 min stirring. The two solutions were combined dropwise, and the pH of the mixture was adjusted to 9.0 ± 0.1 using NaOH (2 mol·L^−1^). Following 1 h of stirring, the mixture underwent hydrothermal treatment at 200 °C for 6 h in a Teflon-lined autoclave. The resultant precipitate was centrifuged, washed with deionized water and ethanol, and dried at 80 °C for 12 h to yield BiVO_4_ powder.

For the TiO_2_-BiVO_4_-M fabrication, 4 mL C_12_H_28_O_4_Ti was blended with 12 mL IPA through 1 h of stirring, followed by the addition of 1 mL glacial acetic acid with subsequent 1 h of mixing. Meanwhile, 0.15 g BiVO_4_ powder was dispersed in 3.2 mL IPA with 0.3 mL deionized water via ultrasonication for 30 min. The BiVO_4_ dispersion was incorporated into the TiO_2_ precursor and stirred for 1 h, and progressively diluted with deionized water until a viscous sol formed. The sol was hydrothermally treated at 200 °C for 2 h. The obtained hydrogel was stirred for 2 h before spin-coating onto a 3 × 3 cm FTO conductive glass using a homogenizer and calcined at 550 °C for 2 h to obtain the TiO_2_-BiVO_4_-M.

In synthesizing TiO_2_-BiVO_4_ layered material (TiO_2_-BiVO_4_-L), BiVO_4_ precursor preparation excluded hydrothermal crystallization. The TiO_2_ sol was formulated by introducing 10 mL C_16_H_36_O_4_Ti into a mixture of 16 mL ethanol and 4 mL glacial acetic acid under continuous agitation. An 80% (*v*/*v*) ethanol solution was incrementally added, with the mixture aged for 24 h to stabilize. The TiO_2_ sol was spin-coated onto cleaned FTO glass, dried at 180 °C, and calcined at 550 °C for 2 h. The BiVO_4_ sol layer was then spin-coated onto the TiO_2_ film, finalized through 550 °C calcination for 2 h.

In the spin-coating process, 0.25 mL of precursor solution was dropped onto FTO glass and allowed to stand for several minutes to ensure sufficient adsorption. The substrate was then rotated in two stages: first at 500 r/s for 10–15 s to spread the solution uniformly, followed by 3000 r/s for 30 s to expel most of the water. This process yielded a reproducible thin film, with the final thickness determined primarily by the number of spin-coating cycles.

### 2.3. Characterization of TiO_2_-BiVO_4_

The morphological characteristics of the prepared materials were observed by the FEI XL 30 ESEM FEG scanning electron microscopy (Hillsboro Field Electron and Ion Company, Hillsboro, OR, USA). The crystalline structure of the prepared materials was characterized by Bruker D8 Advance X-ray diffraction (Berlin Bruker Corporation, Berlin, Germany) with Cu Kα (λ 0.15406 nm) radiation in the range of 10–80° and a scan rate of 0.02°·min^−1^. The element component, chemical state, and valence band binding energy were analyzed by the Thermo Scientific ESCA-LAB 250 X-ray photoelectron spectroscopy (Massachusetts Thermo Fisher Scientific Inc., Waltham, MA, USA). The optical absorption property of the catalyst was recorded by the Shimadzu UV-1800 UV-vis diffuse reflectance spectroscopy (Kyoto Shimadzu Corporation, Kyoto, Japan). Photocurrent–time curve (I-t) and electrochemical impedance spectroscopy (EIS) of the as-prepared materials were measured by a three-electrode electrochemical workstation system (Chenhua CHI-660E, Shanghai, China) equipped with a saturated calomel electrode as reference electrode, a Pt flake as counter electrode and as-prepared catalysts as working electrode.

### 2.4. Photoelectrocatalytic Degradation Experiment

The PEC properties of the as-prepared materials were evaluated through the degradation of RhB. In the PEC degradation study of RhB, 500 mL of a 10 mg·L^−1^ RhB solution was placed in a self-designed quartz reactor. The electric field was applied using an electrochemical workstation, with the voltage set to 2, 4, and 6 V, respectively. Simulated solar light was supplied by a 300 W xenon lamp (Beijing Perfectlight Technology Co., Ltd., Beijing, China, wavelength range 320–780 nm) equipped with a filter to block infrared radiation. The lamp was positioned at distances of 5, 10, or 15 cm from the reactor to adjust the light intensity. The as-prepared material, platinum (Pt) flake, and saturated calomel electrode were used as the working electrode, counter electrode, and reference electrode, respectively. Multiple degradation experiments were performed under various concentrations of RhB (5, 10, and 15 mg·L^−1^), pH values (about 4, 6, 7, and 9), and electrolyte solutions (Na_2_SO_4_ or NaNO_3_ at concentrations of 0.1, 0.25, 0.5, and 1 M) to determine the optimal conditions for PEC degradation of RhB in the presence of TiO_2_-BiVO_4_ and to assess its performance. The RhB solution was stirred for 60 min to ensure adsorption–desorption equilibrium, and samples were taken every 60 min to determine the RhB concentration using a UV-vis absorption spectrometer (Kyoto Shimadzu Corporation, Kyoto, Japan) at 554 nm. In the catalyst recycling experiment, the catalysts were washed several times with a mixture of anhydrous ethanol and ultrapure water UPW after each test, and then dried at 60 °C for 3 h for the next cycle. To minimize errors, all trials were repeated three times.

## 3. Results and Discussion

### 3.1. Optimization of Preparation Process of TiO_2_-BiVO_4_

#### 3.1.1. TiO_2_-BiVO_4_ Composite Material

**Figure 1:**
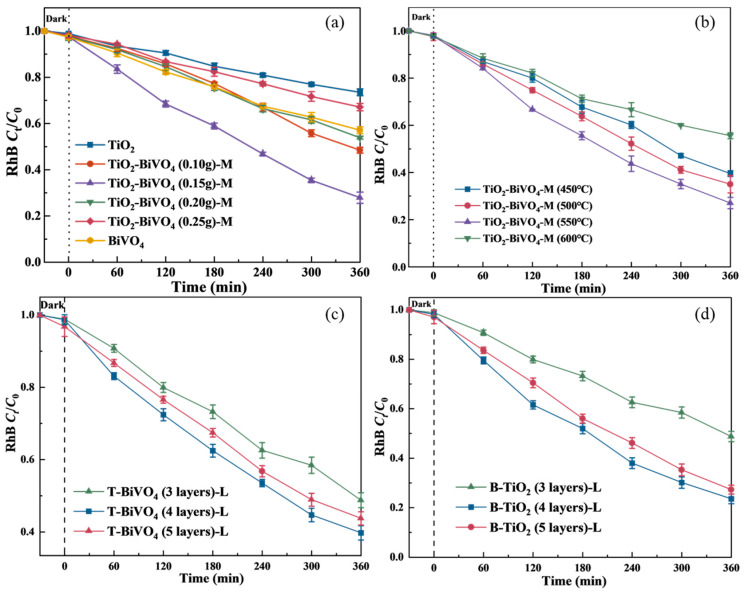
Effects of (**a**) BiVO_4_ loading amount and (**b**) calcination temperature on PEC degradation of RhB in the presence of TiO_2_-BiVO_4_-M, and (**c**) number of BiVO_4_ layers and (**d**) TiO_2_ layers on PEC degradation of RhB in the presence of TiO_2_-BiVO_4_-L (*C*_RhB_ 10 mg·L^−1^, light intensity 14,000 lx, applied bias voltage 2 V, initial pH 7.0 ± 0.1).

In the absence of light, TiO_2_-BiVO_4_ composite materials with different BiVO_4_ loading levels (0, 0.10, 0.15, 0.20, and 0.25 g) could reach adsorption equilibrium within 60 min, with the RhB removal efficiency of less than 3.2% (Figure S1). The photocatalytic results (Figure 1a) showed that after the adsorption equilibrium and simulated sunlight irradiation for 360 min, the degradation efficiencies of pure TiO_2_ and BiVO_4_ on RhB were 26.6% and 42.9%, respectively. While the degradation efficiency of TiO_2_-BiVO_4_ on RhB was obviously increased, and the degradation efficiency on RhB firstly increased and then decreased when the loading amount of BiVO_4_ in the composites increased from 0 g to 0.25 g, and the photocatalytic activities were as follows: TiO_2_-BiVO_4_ (0.15 g) > TiO_2_-BiVO_4_ (0.10 g) > TiO_2_-BiVO_4_ (0.20 g) > TiO_2_-BiVO_4_ (0.25 g), and the degradation efficiencies were 72.1%, 51.6%, 46.2% and 32.9%, respectively. The positive correlation between the degradation rate and the number of active centers of the semiconductor catalyst up to the optimal loading level. An increase in the loading level increases the PEC active centers. However, when the loading level exceeds the optimal value, the catalyst tends to agglomerate, leading to a decline in visible light photocatalytic efficiency [25,26]. Therefore, the optimum loading amount of BiVO_4_ in TiO_2_-BiVO_4_ composites was determined to be 0.15 g.

Calcination temperature is another important factor affecting the properties of TiO_2_-BiVO_4_ composite material. The calcination process not only affects the crystal structure and phase purity of the material, but also influences the specific surface area and pore structure of the material [27]. Appropriate calcination temperatures can promote the tight binding between TiO_2_ and BiVO_4_, enhance the structural stability of the materials, and optimize the transport and separation of photogenerated charges. The degradation curves of RhB (10 mg·L^−1^, pH 7, and applied bias voltage of 2 V) under different calcination temperature conditions are shown in Figure 1b. As the calcination temperature increased, the PEC performances were in the order of TiO_2_-BiVO_4_ (550 °C) > TiO_2_-BiVO_4_ (500 °C) > TiO_2_-BiVO_4_ (450 °C) > TiO_2_-BiVO_4_ (600 °C), with degradation efficiencies of 76.9%, 63.3%, 60.5%, and 44.3%, respectively. Specifically, incomplete crystalline development at low calcination temperature (450 °C) leads to carrier migration limitation, and the increase in calcination temperature (500–550 °C) promotes the crystallization of the anatase phase and enhances charge separation efficiency [28,29]. At higher calcination temperature (600 °C), the crystal structure changes from anatase to rutile, triggering an intensification of carrier complexation, while grain coarsening leads to a decrease in active sites [30].

#### 3.1.2. TiO_2_-BiVO_4_ Layered Material

The layer number of BiVO_4_ significantly impacts the PEC performance of TiO_2_-BiVO_4_ layered structures. By controlling the spin-coating cycles of precursor solutions, the film thickness was precisely regulated, with 3 layers established as the baseline and incrementally increased to 4 and 5 layers for comparative analysis. Experimental results obtained in a pH 7 electrolyte demonstrated that increasing BiVO_4_ layers from 3 to 4 enhanced the RhB degradation efficiency from 51.3% to 60.3% after 360 min, achieving optimal performance (Figure 1c). This improvement originates from extended light absorption pathways and enhanced photon harvesting capability through appropriate layer stacking [31]. However, further increasing to 5 layers resulted in reduced degradation efficiency (55.1%), attributed to compromised light penetration depth and prolonged carrier migration paths. Excessive BiVO_4_ thickness restricts photon access to internal active layers, while lengthened charge transport distances promote recombination losses [32]. Although increased layer numbers theoretically provide additional catalytic sites, over-stacking may induce active site shielding effects that diminish effective reaction interfaces. The synergistic effect of the light absorption enhancement and carrier transport efficiency identifies 4-layer BiVO_4_ as the optimal structural parameter.

The thickness of TiO_2_ layers plays a pivotal role in governing the PEC performance of TiO_2_-BiVO_4_ heterostructures, analogous to the influence of BiVO_4_ layer optimization. Precise matching of spin-coating cycles between TiO_2_ and BiVO_4_ enables spatial modulation of heterojunction interfaces, achieving synergistic effects of broadband light absorption and efficient carrier separation [33]. Experimental results reveal that the 360 min RhB degradation efficiency reached 51.3% when both components were 3-layer structured, consistent with the baseline data from single-variable BiVO_4_ layer studies. Synchronously increasing both layers to 4 significantly enhanced degradation efficiency to 77.5%, primarily attributed to amplified interface effects (Figure 1d). The optimized layer configuration increased heterojunction interface density, thereby improving spatial charge separation [34]. Nevertheless, expanding the layer count to 5 led to a notable decline in degradation efficiency to 72.7%, which can be attributed to several intertwined factors. Specifically, the extended carrier migration paths hindered the efficient charge transport. Additionally, the heightened interfacial defect density facilitated the recombination of photogenerated e^−^/h^+^, thereby reducing the effective charge separation. Furthermore, significant optical shielding effects emerged as the upper TiO_2_ layers impeded photon penetration into the underlying active regions, limiting the light absorption capacity. This multifactorial interplay resulted in the observed reduction in efficiency [35]. Photon transport losses induced by the light-blocking effect of TiO_2_ dominate performance degradation beyond critical layer thresholds. Consequently, the dual 4-layer configuration emerges as the optimal architecture, balancing sufficient photon harvesting, effective interfacial charge separation, and minimized transport losses through systematic design optimization.

### 3.2. Characterization of the Catalysts

#### 3.2.1. Morphological Analysis

**Figure 2:**
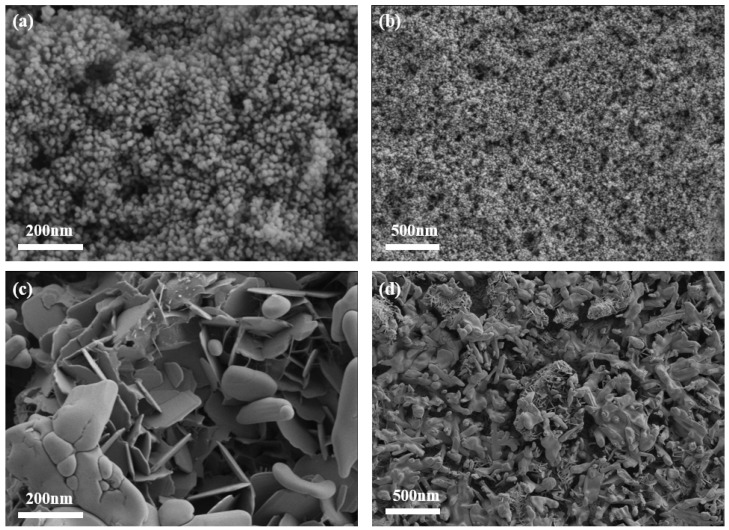
SEM images of (**a**,**b**) TiO_2_-BiVO_4_-M, and (**c**,**d**) TiO_2_-BiVO_4_-L.

The surface morphologies and microstructures of TiO_2_-BiVO_4_-M and TiO_2_-BiVO_4_-L were investigated via SEM images. It can be seen that TiO_2_-BiVO_4_-M (Figure 2a,b) exhibited a granular composition of numerous small particles tightly clustered together. This structure exhibited relatively high porosity, which resulted from the formation of rough, uneven, and irregular surfaces due to the heterogeneous size and distribution of the particles. The voids between these particles were formed by irregular or inconsistent stacking. In contrast, TiO_2_-BiVO_4_-L (Figure 2c,d) consisted of numerous sheets forming lamellar structures. These sheets were stacked on top of each other, each maintaining a relatively flat and well-defined boundary, resulting in a smooth and homogeneous appearance.

**Figure 3:**
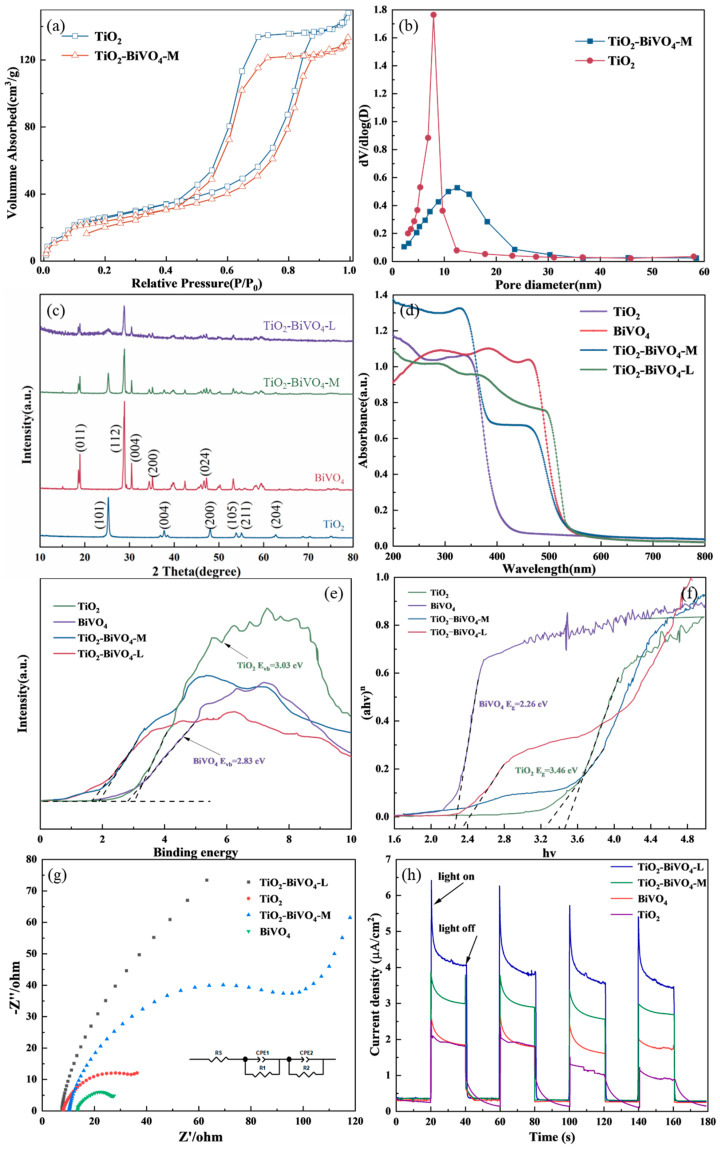
(**a**) N_2_ adsorption–desorption isotherms and (**b**) pore size distribution of TiO_2_ and TiO_2_-BiVO_4_-M, (**c**) XRD patterns and (**d**) UV-Vis DRS of TiO_2_, BiVO_4_, TiO_2_-BiVO_4_-M and TiO_2_-BiVO_4_-L, (**e**) XPS valence band spectra and (**f**) band gaps of TiO_2_ and BiVO_4_, (**g**) EIS plots (The inset is the equivalent circuit diagram) and (**h**) transient photocurrent spectra of TiO_2_, BiVO_4_, TiO_2_-BiVO_4_-M and TiO_2_-BiVO_4_-L.

The adsorption isotherms of both TiO_2_-BiVO_4_-M and TiO_2_ presented the type-I isotherms (Figure 3a). These isotherms showed a rapid increase in the range of P/P_0_ < 0.1, indicating the presence of microporous structures in TiO_2_-BiVO_4_-M. In addition, a type-IV isotherm is typical for mesoporous material, often associated with a hysteresis loop and presents a non-uniform pore structure or irregular pore shape [36]. In the range of 0.2 < P/P_0_ < 1.0, TiO_2_-BiVO_4_-M displayed significant adsorption behavior with a hysteresis loop, consistent with the characteristics of a type IV isotherm. This indicated that TiO_2_-BiVO_4_-M possessed both microporous and mesoporous structures. As shown in Figure 3b, the pore structure of TiO_2_ was relatively stable and narrow, with a pore volume of 0.4467 cm^3^·g^−1^. In contrast, TiO_2_-BiVO_4_-M exhibited a wider distribution of pore sizes and a slightly smaller pore volume of 0.2011 cm^3^·g^−1^. Additionally, the pore structure was more diverse. These features enhanced the light absorption properties of the materials and contributed to improved catalytic activity. It is noteworthy that although TiO_2_-BiVO_4_-M possesses a significantly higher specific surface area (159.6 m^2^·g^−1^) than TiO_2_-BiVO_4_-L (89.0 m^2^·g^−1^), it demonstrates inferior PEC activity for RhB degradation. The high surface area of TiO_2_-BiVO_4_-M originates from its irregular, porous particulate morphology, which may conversely lead to severe light scattering and tortuous charge transport paths, exacerbating charge recombination [37]. In contrast, the well-ordered layered structure of TiO_2_-BiVO_4_-L, while yielding a lower surface area, establishes a more effective heterojunction interface. This architecture significantly enhances the separation and migration of photogenerated charges (Figure 3g,h). Consequently, the overall performance of TiO_2_-BiVO_4_-L surpasses that of the composite material.

#### 3.2.2. Crystal Structure Analysis

The phase structures of the four samples were characterized by XRD (Figure 3c). TiO_2_ presented an anatase-type structure with characteristic diffraction peaks located at 25.3° (101), 37.8° (004), 48.0° (200), 54.4° (105), 55.6° (211), and 62.7° (204) [38]. Similarly, BiVO_4_ presented a monoclinic scheelite-type structure and showed clear diffraction peaks at angles of 18.9° (101), 28.8° (112), 30.6° (004), 34.5° (200), and 47.1° (204) [39]. Comparing the TiO_2_-BiVO_4_-M and TiO_2_-BiVO_4_-L to pure TiO_2_ and BiVO_4_, obvious diffraction peaks corresponding to the anatase and monoclinic scheelite phases were observed in their XRD diffractograms. This indicated that the heterogeneous structure of TiO_2_-BiVO_4_-M and TiO_2_-BiVO_4_-L did not affect the crystalline structure of TiO_2_ and BiVO_4_. It was worth noting that the characteristic peaks around 25.3° for TiO_2_-BiVO_4_-L exhibited obvious weakening and broadening. To quantify any crystallographic changes, the average crystallite size was estimated using the Scherrer equation:

*D* = Kλ/*w*cos*θ* (1)

where *D* represents the crystallite size; K represents the Scherrer constant (0.89); λ denotes the incident X-ray wavelength, determined by the test conditions; for a copper target, λ = 0.15406; *θ* represents the diffraction angle (°); *w* denotes the half-width of the diffraction peak; 2*θ* and *w* are measured by XRD.

The calculated crystallite sizes were 23.3 nm (TiO_2_), 25.7 nm (BiVO_4_), 23.2 nm (TiO_2_-BiVO_4_-M), and 24.1 nm (TiO_2_-BiVO_4_-L), respectively. This indicates that the peak broadening is not primarily due to a reduction in crystallite size.

Instead, the broadening is most likely attributed to the introduction of microstrain within the crystal lattice. The formation of a heterojunction between two distinct materials (TiO_2_ and BiVO_4_) with different crystal structures can induce significant local lattice distortions and defects at the interfaces. This microstrain, a well-known contributor to XRD peak broadening [40], is particularly pronounced in the layered structure (TiO_2_-BiVO_4_-L) due to its extensive and well-defined interfacial contact area. This effect, rather than spatial positioning, is the dominant cause of the observed peak profile changes.

#### 3.2.3. Optical Properties

The UV-vis DRS of several materials is illustrated in Figure 3d. Typically, anatase TiO_2_ has a band gap of approximately 3.2 eV, resulting in strong absorption predominantly in the UV region, with light absorbed below approximately 380 nm [41]. In contrast, BiVO_4_ has a relatively narrow bandgap (2.4 eV), allowing it to absorb a larger portion of the visible spectrum, with an absorption edge around 517 nm. TiO_2_-BiVO_4_-M exhibited photoresponsivity in both the UV and visible regions, due to the heterojunction structure of the two n-type semiconductors, which facilitates the migration of e^−^ from the conductive band (CB) of one semiconductor to the other, while h^+^ migrate in the opposite direction [42]. As a result, the absorption edge of TiO_2_-BiVO_4_-M shifted to lower energies (longer wavelengths) compared to TiO_2_ or BiVO_4_ alone, but the absorption intensity in the visible region was significantly reduced. The TiO_2_-BiVO_4_-L consisted of alternating layers of TiO_2_ and BiVO_4_, leading to a synergistic effect on the light absorption properties of the two materials. This material demonstrates enhanced absorption in both the UV and visible regions, with the visible light absorption extending beyond the range of BiVO_4_ alone, resulting in a broader spectral absorption range.

The energy band structure critically determines semiconductor performance, as evidenced by the XPS valence band spectra in Figure 3e. TiO_2_ exhibited a relatively high valence band potential (*E*_VB, XPS_) of 3.03 eV, while BiVO_4_ exhibited a lower *E*_VB, XPS_ value of 2.83 eV. The corresponding standard hydrogen electrode (*E*_VB, NHE_) can be calculated according to the following formula:

*E*_VB, NHE_ = *φ* + *E*_VB, XPS_ − 4.44 (2)

where *φ* represents the work function of the instrument (4.45 eV) [43]. Thus, the *E*_VB, NHE_ of TiO_2_ and BiVO_4_ is calculated to be 3.04 and 2.84 eV, respectively. According to the formula of the band gap energy (*E_g_*) of the semiconductor:

(*αhν*)*^n^* = *k*(*hν* − *E_g_*) (3)

as shown in Figure 3f, the *E_g_* value of TiO_2_ and BiVO_4_ is estimated to be 3.46 and 2.26 eV, respectively [44]. The conduction band potential (*E*_CB_) of different samples can be calculated according to the empirical formula:

*E*_VB_ = *E*_CB_ + *E_g_* (4)

Thus, the *E*_CB_ of TiO_2_ and BiVO_4_ could be confirmed to be −0.42 and 0.58 eV, respectively.

It is worth noting that the measured band gaps (3.46 eV for TiO_2_ and 2.26 eV for BiVO_4_) show slight deviations from the commonly reported values (~3.2 eV and ~2.4 eV), which may be attributed to differences in calculation methods and material structural; however, these shifts are within the range observed in prior reports and do not affect the type-II band alignment or the proposed charge transfer mechanism [45,46].

#### 3.2.4. Photoelectrochemical Properties

For assessing the injection and separating efficiency of carriers, the transient photocurrent density and electrochemical impedance spectroscopy (EIS) of these samples were measured. The EIS of the photoelectrode is depicted in Figure 3g. The inset showed the equivalent circuit for fitting Nyquist plots, where Rs represents the solution resistance between the reference electrode and the working electrode; CPE is a constant phase angle component that represents the bilayer capacitance; and Rt denotes the charge transfer resistance of the electrode. Generally speaking, the smaller the diameter of the semicircle in the Nyquist plot, the faster the interfacial charge transfer ability [47]. Although the impedance values of the TiO_2_ (21.4 Ω) and BiVO_4_ (18.6 Ω) were much lower than those of the TiO_2_-BiVO_4_-M (62 Ω) and TiO_2_-BiVO_4_-L (241 Ω), the maximum impedance value of these photoelectrodes was approximately 200 Ω, which was relatively low compared to other photoelectrodes.

The photocurrent–time (I-t) curves for TiO_2_, BiVO_4_, TiO_2_-BiVO_4_-M and TiO_2_-BiVO_4_-L samples with typical on-off cycles of intermittent light irradiation are shown in Figure 3h. Comparing the four materials, the photocurrent of TiO_2_-BiVO_4_-L reached 4.1 μA·cm^−2^, which was higher than that of TiO_2_-BiVO_4_-M (3.1 μA·cm^−2^), BiVO_4_ (1.9 μA·cm^−2^), and TiO_2_ (1.85 μA·cm^−2^). The rapid enhancement of the photogenerated current in TiO_2_-BiVO_4_-L may be attributed to the heterogeneous structure formed by TiO_2_ and BiVO_4_, which reduced the band gap and extended the response to the visible region.

### 3.3. Photoelectrocatalytic Degradation of RhB

#### 3.3.1. Optimization of Photoelectrocatalytic Conditions

**Figure 4:**
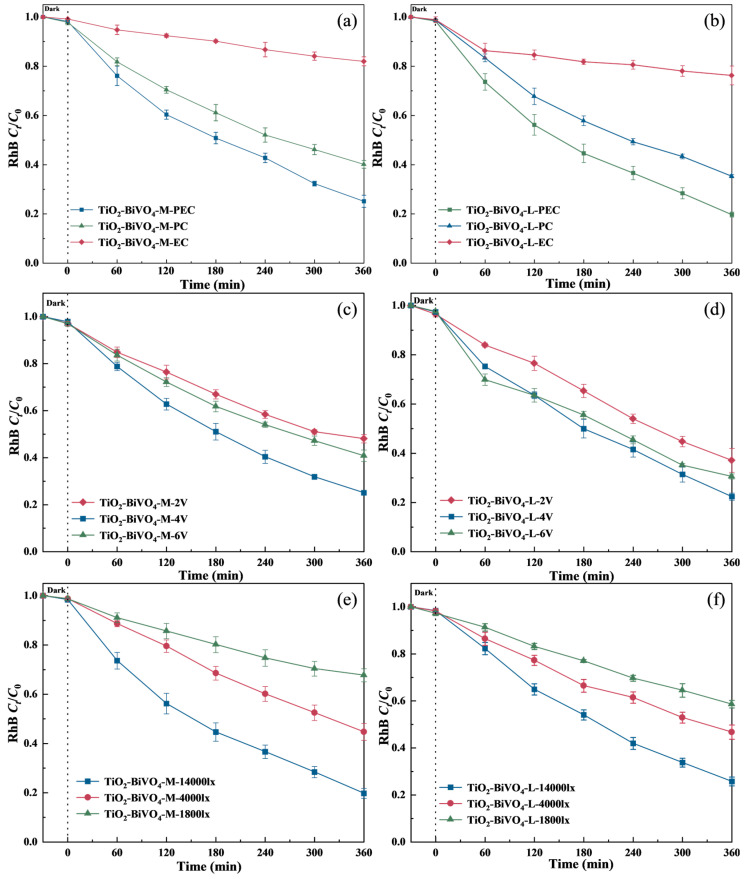
Effect of (**a**,**b**) degradation systems, (**c**,**d**) external bias voltage, and (**e**,**f**) light intensity on RhB degradation (*C*_RhB_ 10 mg·L^−1^, initial pH 7.0 ± 0.1).

The degradation efficiency and reaction kinetic in the presence of as-prepared samples under different systems were investigated by degrading RhB under irradiation for 360 min. As shown in Figure 4a,b, the PEC system exhibited the highest degradation efficiency (74.9% for TiO_2_-BiVO_4_-M and 80.3% for TiO_2_-BiVO_4_-L), which were 15.1% and 15.7% higher compared to the PC system. In contrast, the EC system also catalyzed RhB degradation, but with the lowest efficiency (18.1% for TiO_2_-BiVO_4_-M and 23.8% for TiO_2_-BiVO_4_-L). The limited performance of EC alone highlights the critical role of photoexcitation in generating charge carriers, as purely electrochemical processes lack sufficient redox potential or active species to efficiently degrade complex organic molecules [48]. Meanwhile, the applied bias voltage acting as a driving force to effectively separate photogenerated e^−^/h^+^, thereby prolonging their lifetime and increasing the availability of reactive species for RhB oxidation [49]. The degradation kinetics of RhB under different catalytic systems followed the Langmuir-Hinshelwood first-order kinetic equation (Figure S2). The degradation rate of RhB in the PEC system were 0.00356 mg·L^−1^·min^−1^ (TiO_2_-BiVO_4_-M) and 0.00413 mg·L^−1^·min^−1^ (TiO_2_-BiVO_4_-L), respectively, which were 1.48 and 1.5 times faster than that of the PC system and 6.9 and 6.1 times faster than that of the EC system. These results further demonstrate the higher performance of the PEC system, highlighting the synergistic effect of PC and EC in enhancing the degradation kinetics of RhB compared to standalone photocatalytic or electrochemical methods.

**Figure 5:**
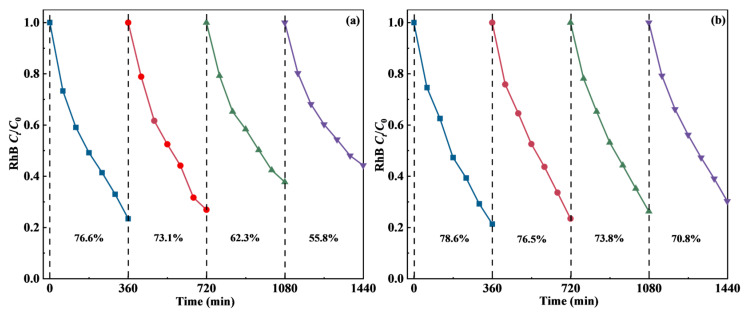
Removal rates for the RhB degradation in the presence of TiO_2_-BiVO_4_ catalysts in four successive cycles: (**a**) TiO_2_-BiVO_4_-M and (**b**) TiO_2_-BiVO_4_-L (*C*_RhB_ 10 mg·L^−1^, light intensity 14,000 lx, applied bias voltage 2 V, initial pH 7.0 ± 0.1).

To investigate the effect of applied bias pressure on the PEC degradation performance of RhB (10 mg·L^−1^, pH 5.8) with the presence of TiO_2_-BiVO_4_ materials, three bias voltages of 2 V, 4 V and 6 V were applied (Figure 4c,d). As the applied bias voltage increased from 2 V to 4 V, the degradation efficiency of RhB increased from 51.9% (TiO_2_-BiVO_4_-M) and 62.9% (TiO_2_-BiVO_4_-L) to 74.9% and 77.6%. However, when the applied bias voltage increased to 6 V, the degradation efficiency decreased to 59.2% and 69.4%, respectively, which was attributed to the limited thickness of the photoelectrode material, where the space charge layer could not exceed the thickness of the semiconductor oxide film. At a fixed light intensity, the number of photogenerated e^−^ reaches an upper limit, so when the applied bias voltage reaches a critical value, the photogenerated carriers have been sufficiently separated, and further voltage increase will not further enhance the catalytic performance [50]. In addition, excessive voltages can lead to certain disadvantages in the charge transfer process, such as electrochemical decomposition of the electrolyte and possible photocorrosion of the composite surfaces [51], as evidenced by reduced stability in recycling tests (Figure 5), which may negatively affect the efficiency of the PEC.

The effect of light intensity in PEC degradation of RhB with the presence of TiO_2_-BiVO_4_ materials is presented in Figure 4e,f. For granular TiO_2_-BiVO_4_-M (Figure 1b and Figure 2a), though surface morphology-induced light scattering can increase effective photon absorption, weak light intensity was not sufficient to fully stimulate the photocatalytic reaction on the material surface, yielding only 41.2% RhB degradation at 1800 lx. Enhanced light intensity (4000 lx) improved efficiency to 53.3%, indicating activated surface reaction sites through increased photon flux that promoted photogenerated carrier generation and separation [52]. Maximum efficiency (74.3%) was achieved at 14,000 lx with stable kinetics. In comparison, TiO_2_-BiVO_4_-L exhibited similar PEC behavior: with increasing light intensity, the degradation efficiency was in the order of 36.4%, 55.4%, and 79.9% (Figure 4f). Under low light intensity, photocarrier recombination dominates interfacial transfer [53]. When the irradiation intensity increased, the layered structure showed higher performance in terms of its ability to capture and convert light energy, with each layer of TiO_2_-BiVO_4_-L potentially participating more fully in the PEC reaction, maximizing the light absorption and catalytic potential of the material.

Although TOC removal was not measured in this study, previous reports on TiO_2_-based and BiVO_2_-based photoelectrocatalysts have shown that high dye decolorization efficiencies generally correspond to TOC removals in the range of 50–70% under similar conditions [40,54,55], suggesting that the present material can also achieve comparable mineralization performance.

#### 3.3.2. Reutilization Performance of TiO_2_-BiVO_4_

The cycling stability of photoanode materials serves as a critical indicator for practical applications, directly determining the long-term operational efficacy of PEC systems. The stability of TiO_2_-BiVO_4_ materials was evaluated by investigating their degradation performance over multiple catalytic cycles (Figure 5). After four catalytic cycles, the degradation rate of RhB in the presence of TiO_2_-BiVO_4_-M within 360 min decreased from 76.6% to 55.8% (Figure 5a). This decline is likely due to partial detachment and erosion of catalyst particles from the electrode surface under the applied bias potential and vigorous stirring during PEC operation. In addition, the accumulation of refractory intermediates may have contributed to active site fouling.

In contrast, the degradation rate of RhB in the presence of TiO_2_-BiVO_4_-L decreased slightly from 78.6% to 70.8% (Figure 5b). Although the degradation efficiency of the materials decreased slightly after each repeated use, the overall decrease in performance was relatively small, sustaining over 70% degradation efficiency after 24 h of continuous operation. This stability can be attributed to its layered architecture, which provides stronger adhesion to the FTO substrate and a more integrated structure that mitigates material loss.

Overall, while TiO_2_-BiVO_4_-M demonstrated higher initial activity, its structural robustness remains a challenge for long-term use. In comparison, TiO_2_-BiVO_4_-L offers a favorable balance of activity and durability, making it more promising for practical PEC applications.

#### 3.3.3. Effects of Water Quality Factors

Water quality factors, such as initial pollutant concentration, pH value, and electrolyte type and concentration, may significantly affect the reaction kinetics during the PEC process.

**Figure 6:**
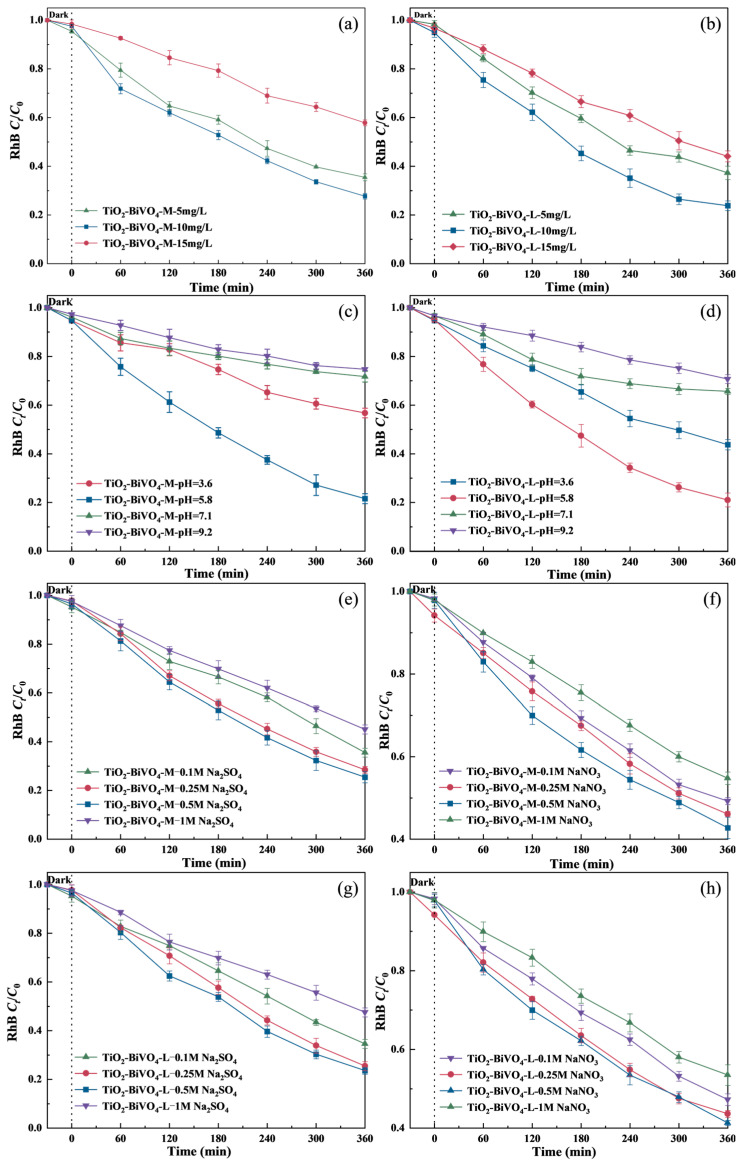
Effect of (**a**,**b**) initial concentrations, (**c**,**d**) pH values, and (**e**–**h**) Na_2_SO_4_ and NaNO_3_ electrolytes and their concentrations on RhB degradation (light intensity 14,000 lx, applied bias voltage 2 V).

Both TiO_2_-BiVO_4_-M and TiO_2_-BiVO_4_-L reached adsorption equilibrium within 60 min (Figure S3) when the initial concentrations of RhB were 5, 10, and 15 mg·L^−1^. Optimal adsorption occurred at 10 mg·L^−1^ of RhB solution, where TiO_2_-BiVO_4_-M and TiO_2_-BiVO_4_-L exhibited adsorption efficiencies of 7.9% and 6.3%, respectively. After 360 min of irradiation, the degradation efficiencies of different initial concentrations of RhB with the presence of TiO_2_-BiVO_4_-M (Figure 6a) were 65.5%, 73.1%, and 43.0%, while those in the presence of TiO_2_-BiVO_4_-L (Figure 6b) were 62.7%, 76.2%, and 55.9%, respectively. At lower initial pollutant concentrations, increasing the concentration could enhance the adsorption of pollutant molecules, thereby better utilizing the activation of the photocatalyst [56]. However, when the initial concentration increased to 15 mg·L^−1^, excessive adsorption of RhB and its intermediates on the catalyst surface led to competition for active sites, reducing the degradation efficiency of PEC [57]. The porous but irregular structure of TiO_2_-BiVO_4_-M suffers from active site occlusion at high pollutant loads. While the higher specific surface area of TiO_2_-BiVO_4_-L is counterbalanced by its ordered pore structure (Figure 2c,d), which mitigates pore blockage by RhB intermediates.

The pH-dependent PEC degradation of RhB is systematically illustrated in Figure 6c,d. Dark adsorption experiments revealed that both TiO_2_-BiVO_4_-M and TiO_2_-BiVO_4_-L achieved adsorption equilibrium within 60 min, exhibiting maximum adsorption efficiencies at pH 5.8 (6.55% and 6.97%, respectively) and minimum values at pH 9.2 (3.02% and 3.62%) (Figure S4). In the PEC degradation study of RhB, the degradation rates of RhB under different pH values with the presence of TiO_2_-BiVO_4_-M within 360 min followed the order of pH 5.8 (78.4%) > pH 3.6 (43.2%) > pH 7.1 (28.3%) > pH 9.2 (25.2%), while TiO_2_-BiVO_4_-L exhibited enhanced efficiencies following the same pH sequence of: pH 5.8 (78.9%) > pH 3.6 (56.2%) > pH 7.1 (34.3%) > pH 9.2 (29.3%). This behavior correlates with the acid-base characteristics of RhB (pK_a_~3.5–5.0) and the surface charge properties of TiO_2_-BiVO_4_. As an amphoteric dye, RhB shows pH-dependent speciation and surface charge characteristics. In acidic conditions, its protonated cationic form dominates due to carboxyl group protonation. In neutral and alkaline environments, deprotonated anionic species are more common [58]. The surface charge of TiO_2_-BiVO_4_ materials is also pH-responsive. TiO_2_ has an isoelectric point around 6.67 [59] and BiVO_4_ has a lower one [60]. Near pH 5.8, the reduced positive surface charge of the catalyst minimizes electrostatic repulsion with cationic RhB species, enhancing adsorption. This is supported by the maximum dark adsorption efficiencies (Figure S4). Under strongly acidic conditions (pH 3.6), although excessive surface protonation causes electrostatic repulsion with RhB cations, reducing adsorption, the high H^+^ concentration promotes h^+^-mediated oxidation and helps generate hydroxyl radical (·OH) through water oxidation [61]. This maintains relatively high degradation efficiency. In neutral and alkaline environments, the intensified electrostatic repulsion between negatively charged catalyst surfaces and anionic RhB molecules greatly limits adsorption. Meanwhile, the elevated pH also suppresses ·OH production via water oxidation pathways [62]. These combined effects account for the sharp decline in degradation efficiency at pH 7.1 and 9.2.

In addition, we explored the effects of two different types of electrolytes (Na_2_SO_4_ and NaNO_3_) and their concentrations (0.1, 0.25, 0.5, and 1 M) on the PEC degradation of RhB with the presence of TiO_2_-BiVO_4_ material. The removal rates of RhB in the presence of TiO_2_-BiVO_4_-M exhibited an initial increasing trend and a decreasing trend with increasing Na_2_SO_4_ concentration. The degradation efficiencies of all concentrations were arranged as follows: 0.5 M Na_2_SO_4_ (74.6%) > 0.25 M Na_2_SO_4_ (71.6%) > 0.1 M Na_2_SO_4_ (64.5%) > 1 M Na_2_SO_4_ (54.9%) (Figure 6e), which was similar with the results of the NaNO_3_ electrolyte system: 0.5 M NaNO_3_ (56.3%) > 0.25 M NaNO_3_ (53.9%) > 0.1 M NaNO_3_ (50.1%) > 1 M NaNO_3_ (43.3%) (Figure 6f). For TiO_2_-BiVO_4_-L, as shown in Figure 6g,h, the PEC degradation results showed a similar trend, but with overall higher efficiency than TiO_2_-BiVO_4_-M. The degradation efficiency was the highest at electrolyte concentration of 0.5 M (77.5% for Na_2_SO_4_ system and 58.8% for NaNO_3_ system) and the lowest at 1 M (52.5% for Na_2_SO_4_ system and 46.6% for NaNO_3_ system). This may be due to the weaker charge shielding effect at lower electrolyte concentrations, which promotes the separation and migration of photogenerated carriers, thus enhancing the PEC degradation process. However, at higher electrolyte concentrations, the strong charge shielding effect may inhibit effective carrier separation, reducing degradation efficiency [63]. Overall, the layered structure may exhibit better PEC degradation performance at different electrolyte concentrations due to the provision of more reactive interfaces and improved charge transport pathways.

Additionally, the steady-state photocurrent–concentration curves of the two electrolyte solutions were investigated (Figure S5). Under identical conditions, the photocurrent intensity of TiO_2_-BiVO_4_ material in Na_2_SO_4_ electrolyte was significantly higher than in NaNO_3_ electrolyte. In addition, as electrolyte concentration increased, the photocurrent for Na_2_SO_4_ rose significantly, while the increase for NaNO_3_ was minimal. Although higher electrolyte concentrations promoted photocurrent generation, the excessive shielding effect may negatively affect overall PEC performance.

In summary, while steady-state photocurrent intensity is directly proportional to electrolyte concentration, optimal PEC degradation performance depends not only on photocurrent generation but also on its effect on the PEC process. Although high electrolyte concentrations produce higher photocurrents, PEC performance is better at lower concentrations due to the shielding effect. Moreover, different electrolyte types can significantly affect PEC performance, with Na_2_SO_4_ outperforming NaNO_3_ in both photocurrent intensity at the same concentration and PEC degradation under identical conditions. Therefore, considering factors such as degradation efficiency and cost, 0.5 M Na_2_SO_4_ was the optimal electrolyte.

### 3.4. Catalytic Mechanisms of TiO_2_-BiVO_4_

#### 3.4.1. Active Species During Photoelectrocatalyzing

**Figure 7:**
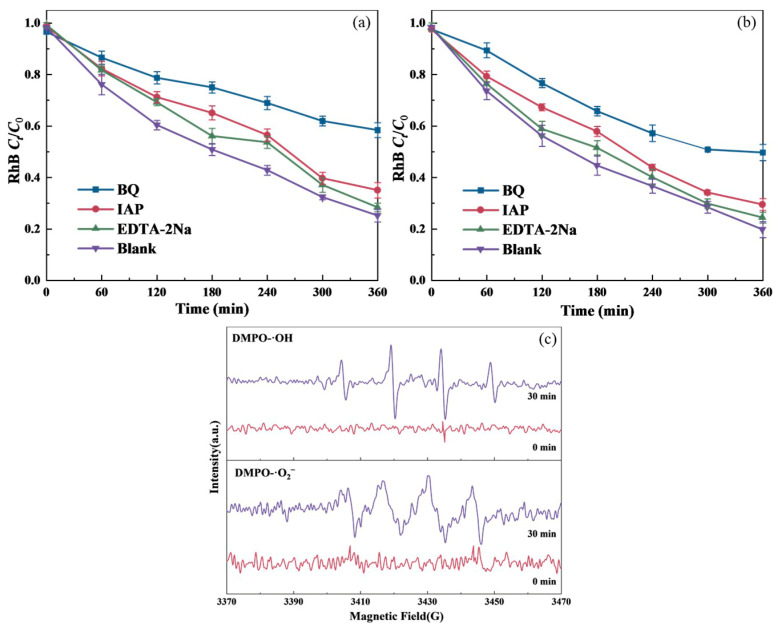
Effect of capture agent on PEC degradation of RhB in the presence of: (**a**) TiO_2_-BiVO_4_-M, (**b**) TiO_2_-BiVO_4_-L, and (**c**) EPR spectra of ·OH and ·O_2_^−^.

To understand the mechanism of BiVO_4_-TiO_2_ degradation of RhB, it is important to discover which reactive species play a major role in the photocatalytic removal process. During the PEC degradation of RhB with the presence of BiVO_4_-TiO_2_, the h^+^, ·OH, and ·O_2_^−^ are eliminated by adding EDTA-2Na, IPA, and BQ as the scavengers, respectively [64,65]. The degradation efficiencies of RhB within 360 min decreased from 74.9% in the absence of scavengers to 71.6%, 64.9%, and 41.6% in the presence of EDTA-2Na, IPA, and BQ, respectively. In the TiO_2_-BiVO_4_-L system, the RhB degradation rates decreased from 80.3% to 75.6%, 70.5% and 50.3% when the mentioned quenchers were added, respectively (Figure 7). The degradation rates of RhB in TiO_2_-BiVO_4_-M and TiO_2_-BiVO_4_-L systems did not decrease significantly with the presence of EDTA-2Na and IPA, suggesting that h^+^ and·OH played a minor role in the situation. BQ obviously hindered the RhB degradation, implying that ·O_2_^−^ was the main active species responsible for the PEC degradation of RhB in the presence of TiO_2_-BiVO_4_. Further analysis of the generation and intensity of the active species was carried out by electron paramagnetic resonance (EPR) spectra (Figure 7c).

EPR experiments using DMPO as spin-trapping agent for ·OH and ·O_2_^−^ [66]. When TiO_2_-BiVO_4_ is illuminated by a 300 W xenon lamp, the characteristic peaks of ·OH and ·O_2_^−^ could be easily detected, which confirmed the presence of ·OH and·O_2_^−^.

#### 3.4.2. Photoelectrocatalytic Mechanism of TiO_2_-BiVO_4_

The highest occupied molecular orbital (HOMO) energy level of RhB is approximately 1.6 V relative to NHE [67], which corresponds to the highest energy state of RhB electrons during electrochemical or photochemical processes. Conversely, the lowest unoccupied molecular orbital (LUMO) energy level of RhB is around −0.6 V (NHE) [68]. This indicates the ability to accept electrons from a molecule, particularly in PEC reactions where electron acceptance is essential for reduction processes.

In terms of the energy levels of the semiconductors, the Fermi energy level (*E*_F_) of TiO_2_ is about 0.1 to 0.2 eV above its *E*_CB_ [69]. As an intrinsic semiconductor, the *E*_F_ of BiVO_4_ is located in the middle of its conduction and valence bands [70]. So, the *E_F_* in pure TiO_2_ and BiVO_4_ was −0.22 and 1.71 eV, respectively. Upon forming the TiO_2_-BiVO_4_ heterostructure, the *E*_F_ of both materials adjusts to reach equilibrium. The higher *E*_F_ of BiVO_4_ shifts downward, while the lower *E*_F_ of TiO_2_ shifts upward. This adjustment facilitates the migration of e^−^ from BiVO_4_ to TiO_2_, while h^+^ is accumulated in BiVO_4_. This anisotropic charge migration not only prolongs the lifetime of e^−^/h^+^ but also enhances their participation in redox reactions.

**Figure 8:**
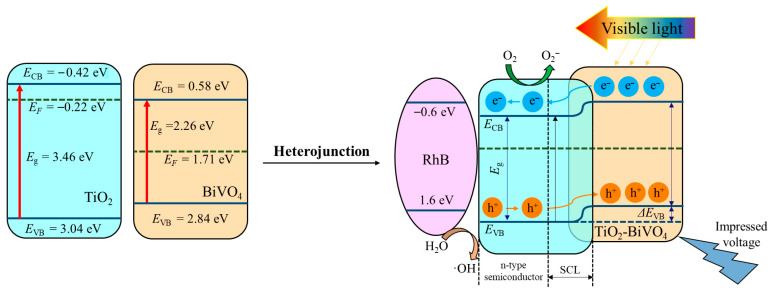
PEC degradation mechanism of RhB in the presence of TiO_2_-BiVO_4_ material.

In the TiO_2_-BiVO_4_ system, PEC activity is predominantly enabled by the formation of a type II heterojunction between TiO_2_ and BiVO_4_. Specifically, the *E*_VB_ of TiO_2_ is more positive than the HOMO of RhB [71] and the potentials of the radical ·OH/OH^−^ (2.38 eV, vs. NHE) and ·OH/H_2_O (2.72 eV, vs. NHE) [72]. This energetic alignment allows photogenerated h^+^ to directly oxidize RhB or react with water to form ·OH. Additionally, since the *E*_CB_ of BiVO_4_ lies lower than that of TiO_2_, e^−^ can transfer from the conduction band of BiVO_4_ to that of TiO_2_, where they are ultimately trapped by oxygen molecules to form ·O_2_^−^ [73]. These reactive oxygen species further participate in the degradation process. The mismatch in valence and conduction band potentials enhances the separation efficiency of e^−^/h^+^, thereby improving the overall photocatalytic performance of the system. The valence band offset in the heterojunction structure effectively minimizes e^−^/h^+^ recombination, thereby boosting the generation of reactive oxygen species (Figure 8).

### 3.5. Degradation Pathways of RhB

In order to investigate the degradation pathway of RhB, we sampled the reaction system of PEC degradation of RhB with the presence of TiO_2_-BiVO_4_ at 0, 30, 60, 120 and 180 min, and carried out UV-visible spectrophotometric detection to infer the degradation pathway of RhB based on the absorption peaks of RhB and its intermediate products at different wavelengths.

**Figure 9:**
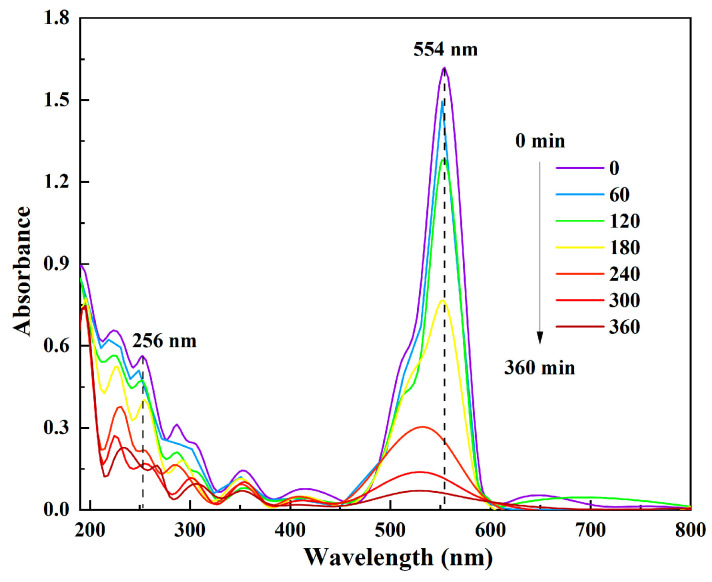
UV-visible light absorption spectrum during the PEC degradation process of RhB.

In the PEC degradation of RhB, the characteristic absorption peak of RhB was mainly located at 554 nm (Figure 9) before the reaction, and the absorption peak continued to decrease with the blue shift phenomenon from the beginning to the end of the PEC reaction in 360 min, which indicated that the structure of RhB was changed during the PEC treatment. At the beginning of the reaction, the absorption peak at 256 nm was also high, and it decreased and blue shifted with the reaction time, which implied that during the PEC degradation of RhB, some side chains of RhB molecules appeared to be stripped of their ethyl groups and certain intermediates were produced. Similarly, Shafaee et al. concluded that the decrease and blue shift in the absorbance of the RhB degradation solution at wavelength 554 nm represent the successive de-ethylation of the RhB molecule and the sequential generation of N,N,N-triethyl rhodamine (539 nm), N,N-diethyl rhodamine (522 nm), N-ethyl RhB (510 nm) and rhodamine (498 nm) [74]. In addition, the degradation of RhB by PEC showed only a decrease in the intensity of the absorption peaks at the late stage of the reaction, and the degradation of RhB was basically completed at 360 min. However, at the later stage, the absorption peaks did not show a blue shift phenomenon, which could be attributed to the breakage of C=C, C=N and C=O in the molecular structure of RhB during the PEC process and the occurrence of ring-opening, which were more conducive to the complete degradation and mineralisation of RhB.

Measurement of UV-Vis absorption spectra was able to demonstrate that intermediate products are produced during the degradation of RhB. In general, the degradation of RhB usually produces a variety of intermediate products, mainly including N-deethylation products, aromatic ring breakage products, carboxylic acids or ammonia, and other nitrogen-containing small molecules. The analysis of reaction intermediates and final products helps to assess the efficiency of the catalytic system and may reveal some details of the reaction process. In order to further explain the degradation pathway of RhB degradation by PEC in the presence of TiO_2_-BiVO_4_ materials, experimental detection was carried out by HPLC-MS/MS, and the intermediate products identified by the analysis are shown in Table S1.

**Figure 10:**
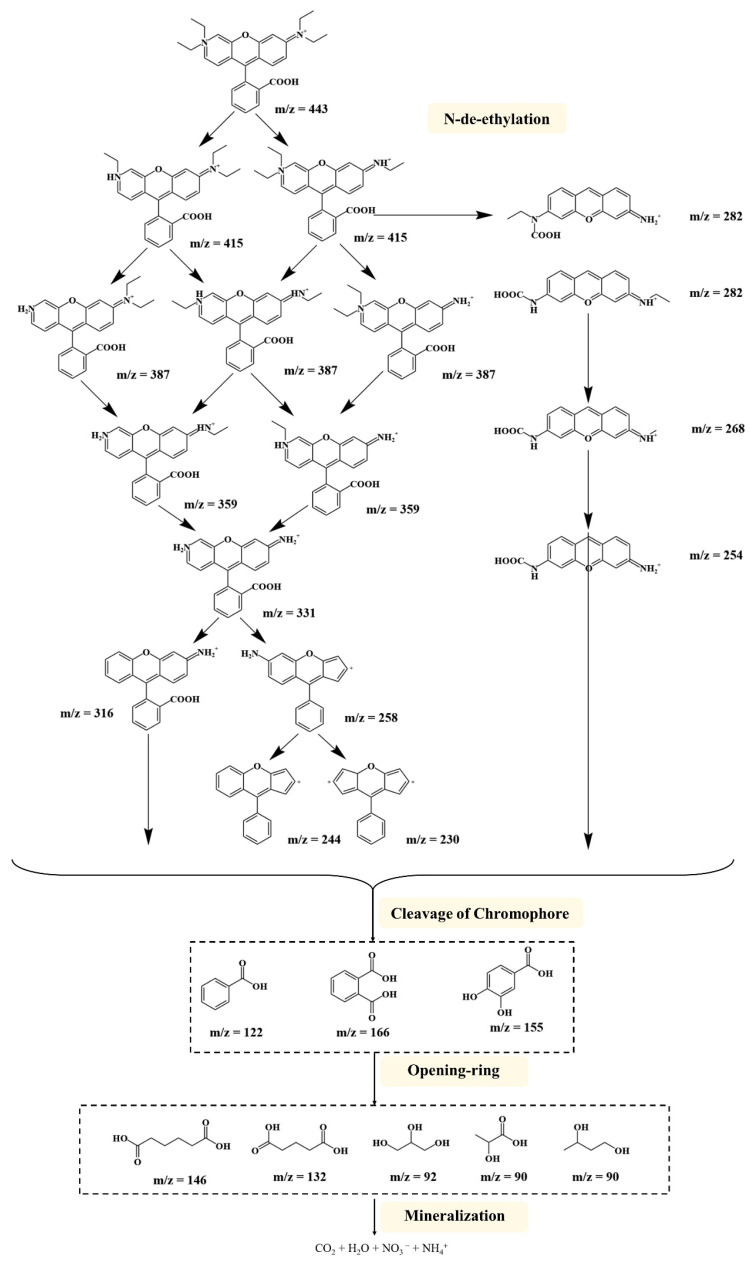
Possible PEC degradation pathways of RhB in the presence of TiO_2_-BiVO_4_ material.

As suggested by Horikoshi et al., the PEC degradation of RhB by photoactive substances (e.g., ·OH and h^+^) can attack the central carbon of RhB, decolourising the dye and further degrading it through the N-deethylation process [75]. In the mass spectra, the main intermediates with m/z values of 443, 415, 387, and 359 were RhB and N-deethylated intermediates, such as N,N-diethyl-N‘-ethyl rhodamine, N,N-diethyl rhodamine, N-ethyl-N’-ethyl rhodamine, N-ethyl rhodamine, N-ethyl rhodamine, and N-ethyl rhodamine. In addition to the N-deethylated intermediates, a number of other intermediates could be identified, i.e., debenzene intermediates and ring-opening intermediates (Figure 10). Ultimately, similar to the literature on the degradation of RhB [76,77,78,79], the oxidation products were mineralised to CO_2_, H_2_O, NO_3_^−^ and NH_4_^+^ [80].

## 4. Conclusions

Against the backdrop of escalating global textile industry emissions, where conventional wastewater treatment methods struggle with persistent organic pollutants due to inefficiency and secondary contamination risks, two distinct TiO_2_-BiVO_4_ heterojunction materials were prepared for the degradation of RhB in water. The fundamental structural differences between these two materials are clearly evidenced by our characterization results. The composite material (TiO_2_-BiVO_4_-M) features an intimately mixed, granular morphology with a high specific surface area (159.6 m^2^·g^−1^) and a porous network comprising both micropores and mesopores. In contrast, the layered material (TiO_2_-BiVO_4_-L) exhibits a well-defined, stratified structure with sequential layers of TiO_2_ and BiVO_4_, resulting in a smoother surface and a lower specific surface area (89.0 m^2^·g^−1^). XRD analysis confirmed that both architectures successfully formed the intended heterojunction without altering the crystalline phases of the individual components, while the notable peak broadening in the layered sample suggests strain induced by the fabrication process.

TiO_2_-BiVO_4_-L exhibited higher degradation efficiency compared to TiO_2_-BiVO_4_-M, attributed to its ordered multilayered configuration, which enhances light absorption across a broader spectral range and provides directional pathways for charge transport. Enhanced stability was also observed in the layered structure, retaining over 70% efficiency after four cycles, which underscores its practical potential for long-term applications. The catalytic mechanism was primarily driven by the formation of a type II heterojunction, where e^−^ were transferred from the conduction band of BiVO_4_ to TiO_2_, while h^+^ accumulated in BiVO_4_, facilitating efficient charge separation. ·O_2_^−^ was identified as the dominant reactive oxygen species responsible for RhB degradation. Degradation pathways of RhB were systematically elucidated, involving sequential deethylation, cleavage of the aromatic chromophore, and subsequent ring-opening mineralization, ultimately leading to the formation of inorganic byproducts such as CO_2_, H_2_O, and NH_4_^+^. This study provides a robust theoretical foundation for the practical application of TiO_2_-BiVO_4_ heterojunctions in treating refractory organic pollutants, highlighting their potential for sustainable wastewater remediation in the printing and dyeing industry. While this study focused on the detailed degradation mechanism of Rhodamine B, future work will expand to include other prevalent dye classes (e.g., azo and reactive dyes) to fully assess the practical potential of the TiO_2_-BiVO_4_ photoelectrode in treating diverse industrial wastewaters.

## Data Availability

The original contributions presented in this study are included in the article/Supplementary Material. Further inquiries can be directed to the corresponding author.

## Supplementary Materials

The following supporting information can be downloaded at: https://www.mdpi.com/article/10.3390/ma18184253/s1, Figure S1: Photoelectroreaction device diagram; Figure S2: Effect of BiVO_4_ loading amounts in the dark adsorption of RhB with the presence of TiO_2_-BiVO_4_-M (*C*_RhB_ 10 mg·L^−1^, initial pH 7.0 ± 0.1); Figure S3. Effect of RhB concentrations in the dark adsorption of RhB with the presence of (a) TiO_2_-BiVO_4_-M and (b) TiO_2_-BiVO_4_-L (*C*_RhB_ 10 mg·L^−1^, initial pH 7.0 ± 0.1); Figure S4. Effect of RhB concentrations in the dark adsorption of RhB with the presence of (a) TiO_2_-BiVO_4_-M and (b) TiO_2_-BiVO_4_-L (*C*_RhB_ 10 mg·L^−1^, initial pH 7.0 ± 0.1); Figure S5. Effect of pH values in the dark adsorption of RhB with the presence of (a) TiO_2_-BiVO_4_-M and (b) TiO_2_-BiVO_4_-L (*C*_RhB_ 10 mg·L^−1^, initial pH 7.0 ± 0.1); Figure S6. Steady-state photocurrent of TiO_2_-BiVO_4_ materials electrode oxidizing organic matter in different electrolyte solutions: (a) TiO_2_-BiVO_4_-M and (b) TiO_2_-BiVO_4_-L; Figure S7. UV-visible light absorption spectrum during the PEC degradation process of RhB; Table S1: Main degradation intermediates of RhB.
