# Supplementary material for: Photoelectrocatalytic Degradation of Rhodamine B in the Presence of TiO2-BiVO4
Source: Materials (Basel). 2025 Sep 11;18(18):4253. doi: 10.3390/ma18184253 (PMC12471973; doi:10.3390/ma18184253)
Supplement: Supplementary file 1 [file materials-18-04253-s001.zip › materials-3841819-supplementary.pdf]

Photoelectrocatalytic degradation of Rhodamine B in the presence of TiO_2-$BiVO_4$

Anli Sun[+], Chao Kong[+], Jie Wang, Beihai Zhou, Huilun Chen, Rongfang Yuan*

Beijing Key Laboratory of Resource-oriented Treatment of Industrial Pollutants, Department of Environmental Science and Engineering, School of Energy and Environmental Engineering, University of Science and Technology Beijing, Beijing 100083, China.

[+] These authors contributed equally to this work.

Corresponding author: yuanrongfang@ustb.edu.cn (R. Yuan).

Figures: S1-S7, Page 2-8;

Table: S1, Page 8-10.

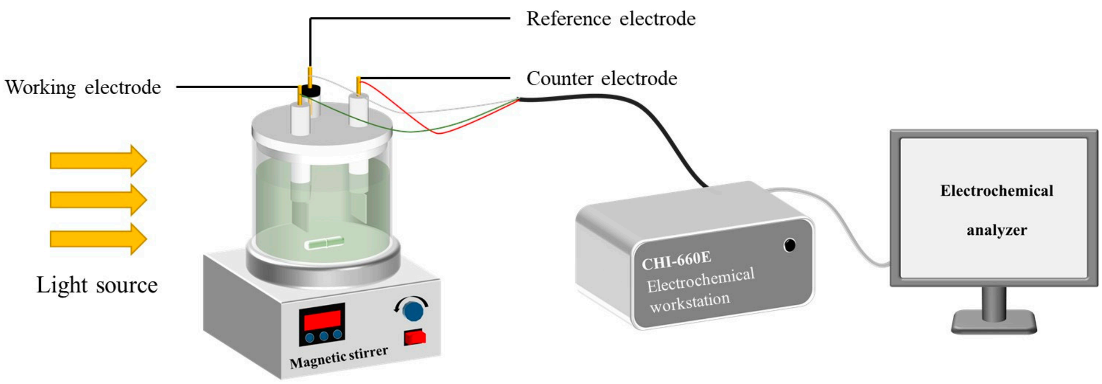

Fig. S1. Photoelectroreaction device diagram.

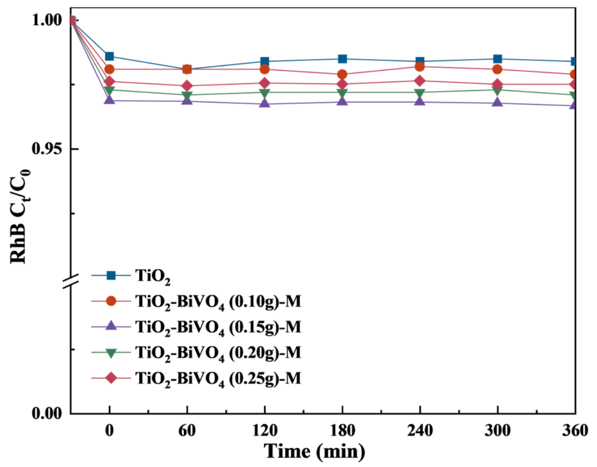

Fig. S2. Effect of $BiVO_4$ loading amounts in the dark adsorption of RhB with the presence of TiO_2-$BiVO_4$-M (C_{RhB} 10 mg·L^{-1}, initial pH 7.0±0.1).

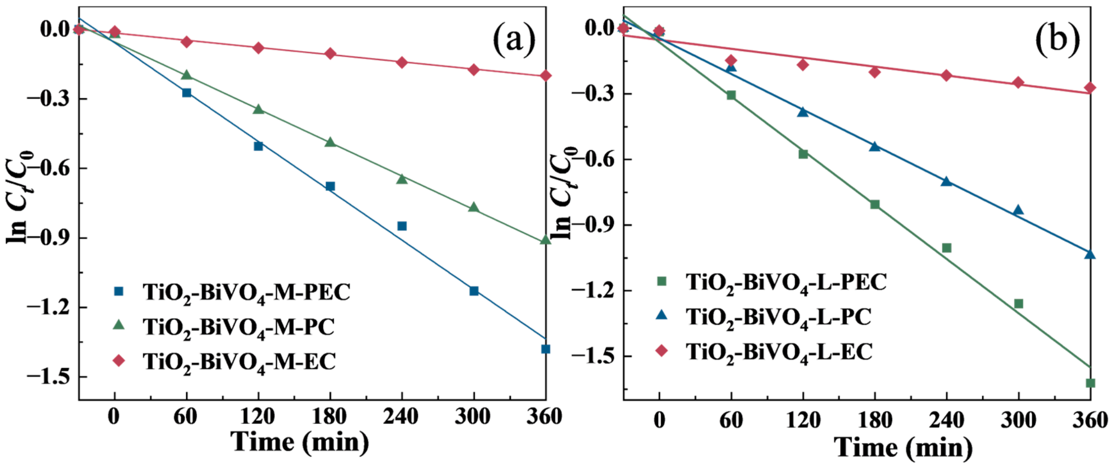

Fig. S3. Effect of RhB concentrations in the dark adsorption of RhB with the presence of (a) TiO_2-$BiVO_4$-M and (b) TiO_2-$BiVO_4$-L (C_{RhB} 10 mg·L^{-1}, initial pH 7.0±0.1).

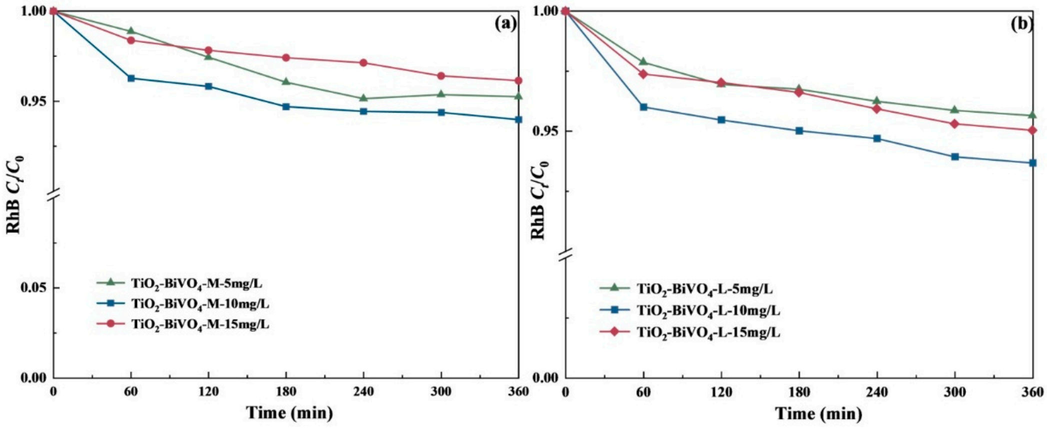

Fig. S4. Effect of RhB concentrations in the dark adsorption of RhB with the presence of (a) TiO_2-$BiVO_4$-M and (b) TiO_2-$BiVO_4$-L (C_{RhB} 10 mg·L^{-1}, initial pH 7.0±0.1).

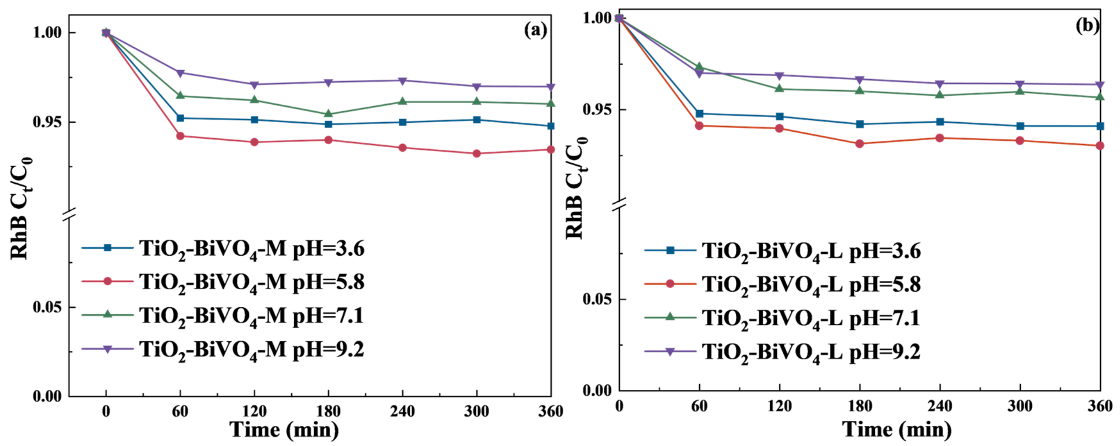

Fig. S5. Effect of pH values in the dark adsorption of RhB with the presence of (a) TiO_2-$BiVO_4$-M and (b) TiO_2-$BiVO_4$-L (C_{RhB} 10 mg·L^{-1}, initial pH 7.0±0.1).

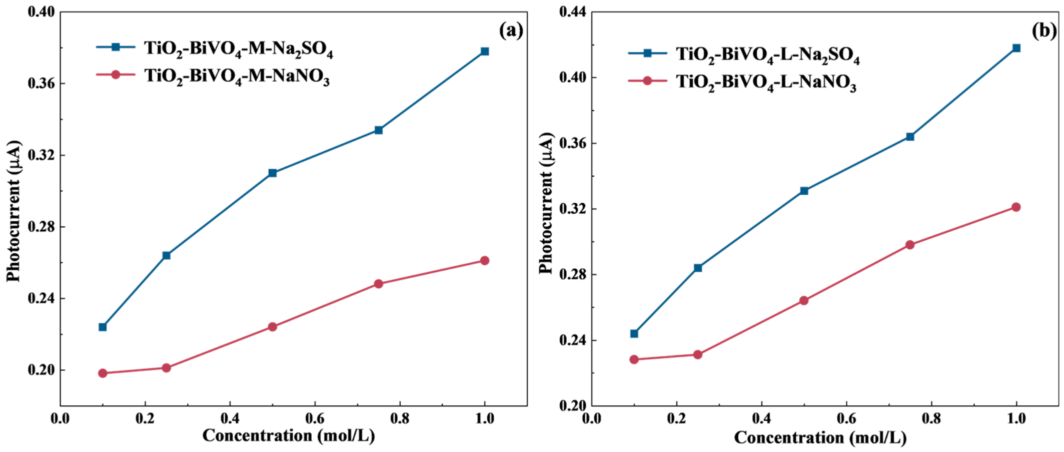

Fig. S6. Steady-state photocurrent of TiO_2-$BiVO_4$ materials electrode oxidizing organic matter in different electrolyte solutions: (a) TiO_2-$BiVO_4$-M and (b) TiO_2-$BiVO_4$-L.

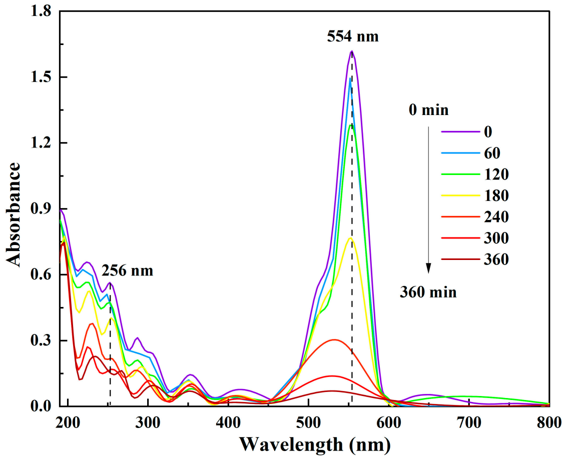

Fig. S7. UV-visible light absorption spectrum during the PEC degradation process of RhB.

Table S1. Main degradation intermediates of RhB.

Retention time (min)	m/z	Molecular formula	Chemical structure
6.21	443	$C_{28}H_{31}N_2O_3$	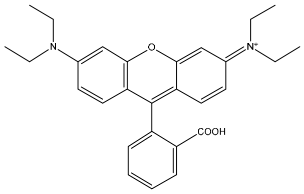
5.65	415	$C_{26}H_{27}N_2O_3$	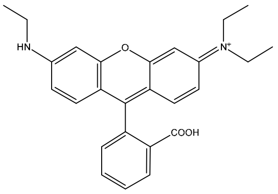
5.65	415	$C_{26}H_{27}N_2O_2$	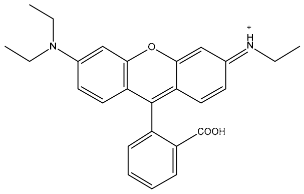
4.78	387	$C_{24}H_{23}N_2O_3$	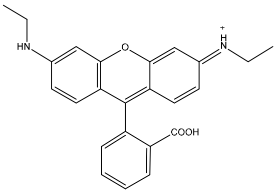
4.78	387	$C_{24}H_{23}N_2O_3$	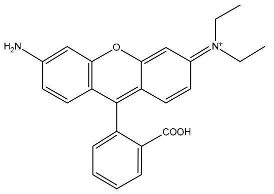
4.78	387	$C_{24}H_{23}N_2O_3$	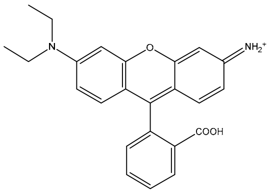
5.12	359	$C_{22}H_{19}N_2O_3$	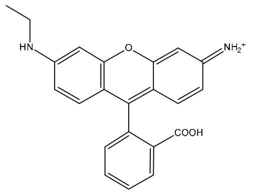

5.12	359	$C_{22}H_{19}N_2O_3$	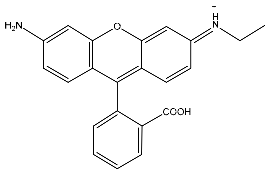
5.80	331	$C_{20}H_{15}N_2O_3$	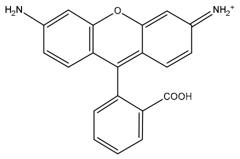
6.21	316	$C_{20}H_{14}NO_3$	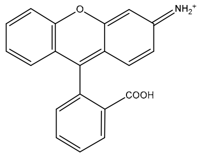
5.39	282	$C_{16}H_{15}N_2O_3$	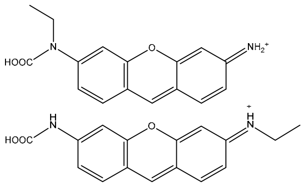
17.41	268	$C_{15}H_{13}N_2O_3$	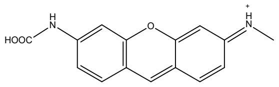
8.06	258	$C_{18}H_{13}NO$	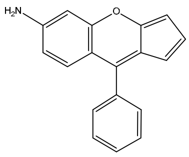
10.06	254	$C_{14}H_{11}N_2O_3$	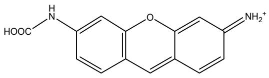
7.34	244	$C_{18}H_{12}O$	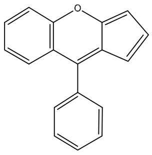
14.68	230	$C_{17}H_{11}O$	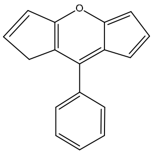
13.66	166	$C_8H_6O_4$	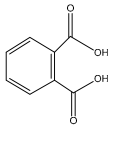
9.14	155	$C_7H_6O_4$	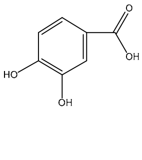

10.54	146	$C_6H_{10}O_4$	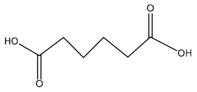
9.70	132	$C_5H_8O_4$	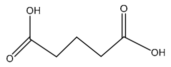
11.93	122	$C_7H_6O_2$	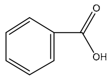
5.45	92	$C_3H_8O_3$	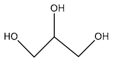
3.47	90	$C_3H_6O_3$	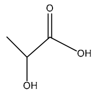
5.44	90	$C_4H_{10}O_2$	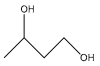
